# Efficiency of Iranian Hospitals Before and After Health Sector Evolution Plan: A Systematic Review and Meta-Analysis Study

**DOI:** 10.3389/fpubh.2021.727669

**Published:** 2021-11-25

**Authors:** Saeed Amini, Behzad Karami Matin, Mojtaba Didehdar, Ali Alimohammadi, Yahya Salimi, Mohammadreza Amiresmaili, Ali Kazemi-Karyani

**Affiliations:** ^1^Department of Health Services Management, Arak University of Medical Sciences, School of Health, Arak, Iran; ^2^Molecular and Medicine Research Center, Khomein University of Medical Sciences, Khomein, Iran; ^3^Research Center for Environmental Determinants of Health, Health Institute, Kermanshah University of Medical Sciences, Kermanshah, Iran; ^4^Department of Medical Parasitology and Mycology, School of Medicine, Arak University of Medical Sciences, Arak, Iran; ^5^Department of Forensic Medicine, School of Medicine, Arak University of Medical Sciences, Arak, Iran; ^6^Social Development and Health Promotion Research Center, Health Institute, Kermanshah University of Medical Sciences, Kermanshah, Iran; ^7^Department of Health Management, Policy Making and Economics, Kerman University of Medical Sciences, Kerman, Iran

**Keywords:** efficiency, hospital, ownership, costs, Iran

## Abstract

**Purpose:** Aging, chronic diseases, and development of expensive and advanced technologies has increased hospitals costs which have necessitated their efficiency in utilization of resources. This systematic review and meta-analysis study has assessed the efficiency of Iranian hospitals before and after the 2011 Health Sector Evolution Plan (HSEP).

**Methods:** Internal and external databases were searched using specified keywords without considering time limitations. The retrieved articles were entered into EndNote considering inclusion and exclusion criteria, and the final analysis was performed after removing duplicates. Heterogeneity between the studies was assessed using Q and I^2^ tests. A forest plot with 95% confidence intervals (CI) was used to calculate different types of efficiency. The data were analyzed using STATA 14.

**Results:** Random pooled estimation of hospitals technical, managerial, and scale efficiencies were 0.84 (95%CI = 0.78, 0.52), 0.9 (95%CI = 0.85, 0.94), and 0.88 (95%CI = 0.84, 0.91), respectively. Sub-group analysis on the basis of study year (before and after HSEP in 2011) indicated that random pool estimation of technical (0.86), managerial (0.91), and scale (0.90) efficiencies of Iranian hospitals for 2011 and before were better than technical (0.78), managerial (0.86), and scale (0.74) efficiencies after 2011.

**Conclusion:** Type of hospital ownership was effective on hospital efficiency. However, HSEP has not improved hospital efficiency, so it is necessary for future national plans to consider all aspects.

## Introduction

Hospitals have an undeniable role in providing healthcare services to society but their increasing costs have become an important challenge for many countries. In other words, utilization of technologies and new methods of diagnosis and treatment of diseases and also increasing numbers of elderly citizens, increasing chronic diseases, increasing demands for healthcare services and specialists, and hospital errors have increased health system costs (1, 2). Because of these issues and problems, hospitals always encounter human and financial resource constraints which have necessitated efficiency in consuming resources more than ever (3).

The efficiency concept has been created from the combination of technical and allocative efficiencies. Technical efficiency means using the lowest amount of input to produce a specified amount of output or using a specified amount of input to produce more output. Allocative efficiency means using the correct amount of input in terms of prices to produce a specified amount of output. Technical efficiency, on the other hand, was created by multiplying scale efficiency and managerial efficiency. Scale efficiency is the ability of an organization unit to perform in or near the most profitable scale to prevent loss in resources. Lastly, managerial efficiency means hard working, correct policymaking, application of the correct number of employees, and the correct combination of production factors (4).

One of the most widely used methods in assessment of different decision-making units (DMUs) such as hospitals and other organizations in terms of the components of efficiency (e.g., technical, scale, and managerial efficiency) is the data envelopment analysis (DEA) method. It is possible, through this method, to create a logical framework to distribute human and financial resources between different wards and sections of studied organizations (5). The DEA method, as a non-parametric programming technique, has been used since the mid 1980s to measure DMU efficiency (6). In other words, linear and multiple programming models are used in this method to assess the relative efficiency of a field, section, unit, or an organization, as a DMU, using multiple input and output indices (7).

Numerous studies have assessed the efficiency of hospital efficiency using the DEA method. These studies can be divided into four categories. In the first category, the efficiency of university, teaching, and public hospitals, as the main providers of healthcare and therapeutic services, has been assessed in studies by Kalhor et al. (8) and Nabi lou et al. (9). In the second category, the efficiency of private hospitals has been studied and their efficiency has been compared with the first-category hospitals (10, 11). The third category includes studies on hospitals affiliated with special entities such as Social Security Organization (12, 13) and Armed Forces (14). The last category measures the efficiency of hospital wards such as radiology (15), dentistry (4), intensive care unit (16), and emergency (17) departments. Because the latter category studies wards of hospitals rather than the hospitals in their entirety and also have not assessed the technical, managerial, and scale efficiency of hospitals wholly, this category was excluded from the current study.

Although many studies have assessed the efficiency of hospitals using the DEA method in Iran, there has been no systematic review and meta-analysis study in this regard to present the final situation of hospital efficiency in Iran. By determining technical, managerial, and scale efficiency of Iranian hospitals, policymakers and planners can improve hospital efficiency through improving distribution and consumption of resources.

The extensive review of the literature by the authors of the current study has resulted in four systematic review and meta-analysis studies on Iranian hospital efficiency using the DEA method. The first study assessed studies in terms of the provinces where they were performed, whether they were input- or output-oriented, and whether they were fixed or variable return to scale models (18). The researchers in another two systematic and meta-analysis studies discussed the methods used to assess hospital efficiency (19, 20). The last study only included a small number of studies on hospital efficiency and did not mention the efficiency subdimensions namely scale, managerial, and technical efficiency (21). As previous systematic review and meta-analysis studies have not assessed hospital efficiency using its subcategories, the current study assessed technical, managerial, and scale efficiency of hospitals through systematic review and meta-analysis.

Regarding PICOS framework or questions, the study included hospitals in Iran which had previously had their efficiency assessed and were entered into the study depending on the inclusion and exclusion criteria. The intervention framework was the assessment of the effect of HSEP on hospital efficiency, comparisons included comparing hospital efficiency before and after HSEP, outcomes included the amount of hospital efficiency, and finally the study design included assessment of hospital efficiency through systematic review and meta-analysis.

## Materials and Methods

### Search Strategy

The international databases of the Institute for Scientific Information (ISI), PubMed, Scopus, Google Scholar, and Persian databases of Scientific Information Database (SID), Magiran, and Barakat were searched using the combination of “efficiency,” “hospital,” “data envelopment analysis,” “DEA,” and “Iran” keywords in 2018. The references of the retrieved articles were searched to increase the study credibility and precision.

### Inclusion and Exclusion Criteria

All published Persian and English language articles about hospital efficiency with a score between 8 and 12 were entered into the study without considering a time limit. If several formats of a research were published (such as a book, article, report, and so on), only one of them was entered into the study. Input-oriented studies were entered into the study. Short reports, letters to editors or editorial comments, one study that was available in two languages, studies on health care facilities other than hospitals, and studies on internal parts of hospitals were removed from the study. Two researchers assessed and extracted data from the studies independently and a third researcher resolved disagreements if they appeared.

This systematic review and meta-analysis utilized Preferred Reporting Items for Systematic Reviews and Meta-Analyses (PRISMA) guidelines to minimize potential sources of bias (22).

### Data Collection

A researcher-made checklist in an Excel spreadsheet was created to extract the studies' data including the first author name, year of data collection, place of study, language, sample size, and the score of technical, managerial, and scale efficiency. Another checklist designed previously, whose credibility has been proved by numerous studies, was used to assesses the studies' quality (19, 21). This checklist includes 12 questions regarding the study aim, method, data collection, sample size, and study population. Each question has the score between 0 and 1 and the score for each study is calculated by summing the scores of questions. So that the studies with scores between 8 and 12 were entered into the final analysis. The study protocol was approved by the Ethical Committee of Kerman University of Medical Sciences.

### Data Analysis

Efficiency types were considered as a proportion in this study. Therefore, the numerator was the sum of technical, managerial, and efficiency scores and the denominator was the number of study hospitals. Heterogeneity between the studies was assessed using Q and I^2^ tests. A *P*-value lower than 0.05 for the Q-test and an I^2^ higher than 50% were considered as the measures of studies' heterogeneity. Because the studies were heterogeneous, the random effect model was used to estimate hospital efficiency. A forest plot with 95% confidence intervals (CI) was used to calculate different types of efficiency. Egger's and Begg's tests were used to assess publication bias. In order to assess the effect of the 2011 Iran Health Sector Evolution Plan (HSEP) (23) on hospital efficiency, the studies before and after it were compared. The data were entered into Excel 2016 to be edited and then transmitted and analyzed using STATA v.14.2.

## Results

**Figure 1:**
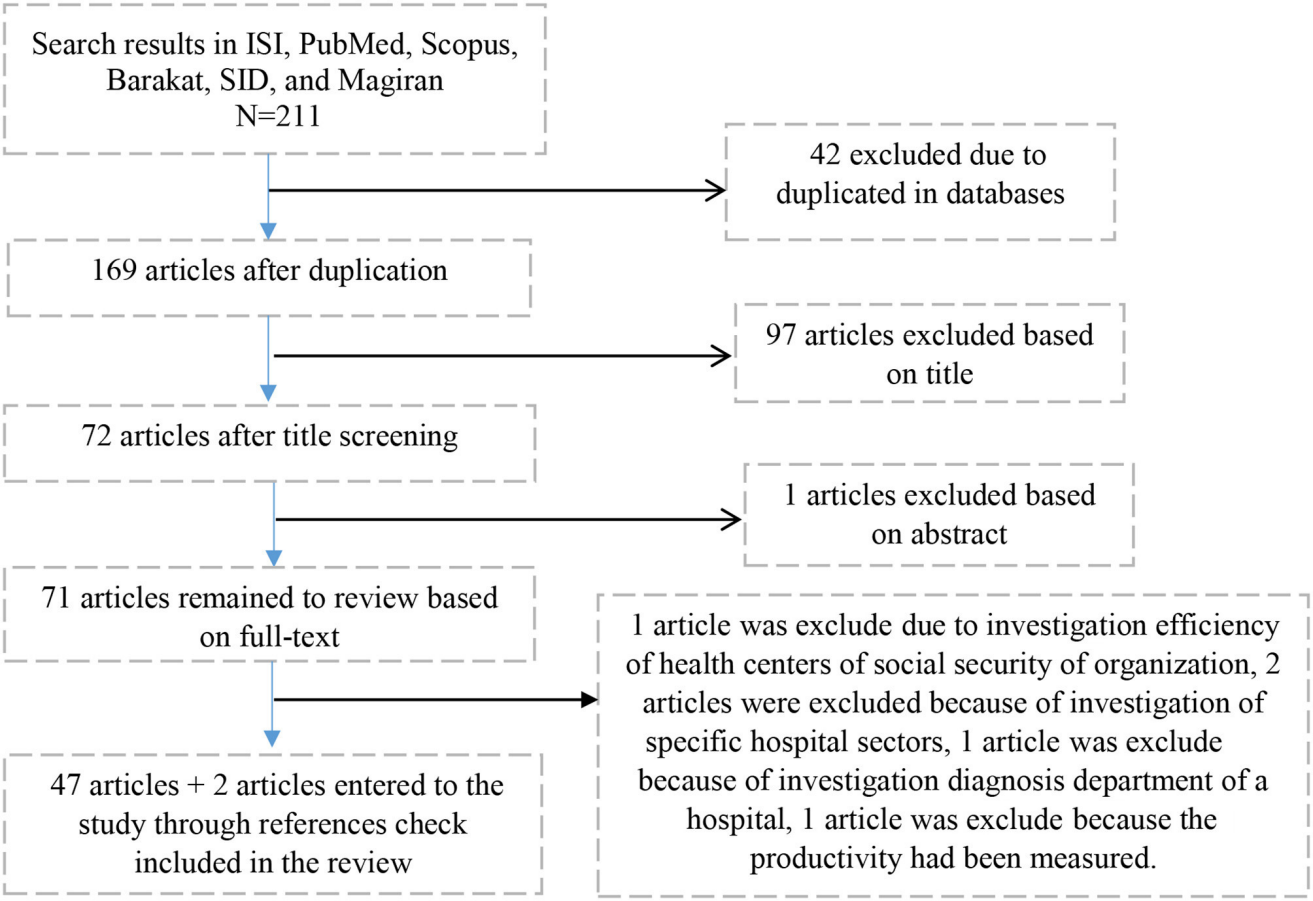
Flow chart of systematic search and studies selection.

Each one of the scientific databases were searched on the basis of a recommended search strategy by the databases themselves using defined keywords. For example, in the PubMed database, 23 articles were retrieved after placing the search query. Search query used for PubMed was: (((data envelopment analysis) OR DEA) AND hospital) AND Iran))). Among retrieved articles, nine articles had assessed efficiency in other areas such as radiology units, intensive care units, and health centers which were excluded from the study. So, finally 14 articles from the PubMed database were entered into the EndNote software. In other databases, after adjusting the search query on the basis of the database guide and then removing unrelated retrieved articles through reading titles, abstracts, and texts, 25 articles from Scopus, 41 from Google Scholar, 8 from Web of Science, 16 from Barakat, 14 from Magiran, and 7 from SID were entered into the EndNote software. After combination of these articles in the EndNote software and removing duplicate articles, 47 final articles remained. Also, the assessment of references of these articles resulted in two new articles. In this way, 49 articles were entered into the final step of the systematic review and meta-analysis. Twelve articles (24.48%) of these were in the Persian language and the remaining were in the English language. A PRISMA flow chart of the study retrieval and selection process with reasons for exclusion at each stage is provided in Figure 1.

By attention that some studies have reported efficiency in several forms or in different scenarios and different inputs were used in them, we considered them as separated studies. In this regard, studies of Hatam et al., Karimi et al., Salehzade et al., Raeisian et al., Firouzi Jahantigh et al., and Sheikhzadeh et al. were each considered as two separated studies. Studies of Joshan et al. and Asadi et al. were each considered as three studies and lastly studies of Fazeli et al. and Mahfoozpor et al. were each considered as four studies. The average number of hospitals entered into the studies was 17.59 hospitals. The lowest and the highest number of hospitals belonged to Rezapour et al. with 4 hospitals and Aboulhalaj et al. with 122 hospitals, respectively.

As mentioned before, each type of efficiency was entered into the meta-analysis separately, so that 50 studies for technical efficiency, 36 studies for managerial efficiency, and 41 studies for scale efficiency had entry requirements to the analysis.

The studies were performed from 1996 to 2016. After performing all the steps of study selection, 49 articles were entered into the final phase of the study. The number of hospitals assessed in these articles ranged from 4 to 122. The inputs considered in the studies included number of beds, number of operation rooms, physicians, nurses, support forces and other human resources, costs, education hours, and working days. The outputs were number of surgeries, outpatients, occupancy rate, bed days, admission, inpatients, surgeries, emergencies, bed turnover, mean patient stay, hospital income, bed occupancy rate, SERVQUAL score, number of clinical, paraclinical, and outpatient services, number of discharged patients, number of contracted insurance companies, access to emergency room, confronted with hospital infections, anesthesia problems, employee consent, active to fixed bed ratio, number of deaths, and patient-day. Two studies assessed charity hospitals, four studies assessed private hospitals, and five studies assessed Social Security Organization (SSO) hospitals. The remaining studies assessed hospitals affiliated with universities of medical sciences belonging to the Iran Ministry of Health (Table 1).

**Table 1:** Characteristics of the studies included in the systematic review and meta-analysis.

**Row**	**Authors**	**Years of data collection**	**Language**	**Location**	**Affiliation of hospitals**	**Number of hospital**	**Inputs**	**Outputs**	**Model of DEA**
1	Joshan et al. (24)	2011–12	Persian	Tehran	TUMS	14	Number of beds, operation rooms, physicians, nurses, and support forces	Number of surgeries, outpatients, patients, bed occupancy rate, bed day, and admission-inpatient rate	VRS, input-oriented
2	Joshan et al. (24)	2011–12	Persian	Tehran	IUMS	8	Number of beds, operation rooms, physicians, nurses, and support forces	Number of surgeries, outpatients, patients, bed occupancy rate, bed day, and admission-inpatient rate	VRS, input-oriented
3	Joshan et al. (24)	2011–12	Persian	Tehran	SBMU	10	Number of beds, operation rooms, physicians, nurses, and support forces	Number of surgeries, outpatients, patients, bed occupancy rate, bed day, and admission-inpatient rate	VRS, input-oriented
4	Sepehrdost et al. (25)	2007–08	Persian	Iran	SSO	28	Number of medical staff, nurses, other sources, and active beds	Number of outpatients, inpatients, surgeries, and bed turnover	CRS, input-oriented
5	Sepehrdost et al. (25)	2007–08	Persian	Iran	SSO	37	Number of medical staff, nurses, other sources, and active beds	Number of outpatients, inpatients, surgeries, and bed turnover	CRS, input-oriented
6	Ghaderi et al. (26)	2005–09	Persian	Tehran & Alborz	IUMS	26	Number of beds, nurses, and others	Number of surgeries, outpatients, hospitalization day, and occupied bed day ratios	VRS, input-oriented
7	Karimi et al. (27)	2005–06	Persian	Isfahan	MUI	23	Number of physicians, nurses, and beds	Mean patient stay, bed turnover, bed occupancy, number of outpatients, and hospital income	VRS, input-oriented
8	Mohammadi Ardakani et al. (28)	2004–06	Persian	Yazd	SSO	12	Number of physicians, paramedics, and active beds	Number of inpatients and outpatients, occupied bed day	Input- and output-oriented
9	Pourreza et al. (29)	1996–98	Persian	Tehran	TUMS	12	Number of beds, nurses, physicians, and others	Number of outpatients, hospitalization-day, number of surgeries	VRS, input-oriented
10	Aboulhalaj et al. (30)	2009	Persian	Iran	MHH	122	Number of beds, physicians, paramedics, and others	Income and admission	VRS, input-oriented
11	Salehzade et al. (31)	2007	Persian	Qom	MUQ & Self-administered	8	Number of physicians, paramedics, and active beds	Number of outpatients and inpatients	VRS, input-oriented
12	Salehzade et al. (31)	2007	Persian	Qom	MUQ & Self-administered	8	Number of physicians, paramedics, and active beds	Number of inpatients and outpatients	CRS, input-oriented
13	Asadi et al. (32)	2008	Persian	Yazd	SSO	13	Costs, education hours, and staff relocation	SERVQUAL score, ratios, outpatient, inpatient, and emergency patients to physicians score	Input- and output-oriented (input-oriented)
14	Askari et al. (33)	2001–08	Persian	Yazd	SSO	13	Number of active beds, nurses, physicians, and others	Number of inpatients, bed occupancies, surgeries	VRS, input-oriented
15	Ilbeigi et al. (34)	2009	Persian	Mashhad	MUMS	17	Number of beds, physicians, nurses, paraclinical staff, and support forces	Inpatient-bed day, outpatients, and surgeries	VRS_CRS (VRS), input-oriented
16	Rahimi et al. (35)	2009	Persian	W. Azarbaijan	UMSU	23	Number of beds, physicians, and others	Occupied bed-day, outpatient admission	VRS, input-oriented
17	Najarzadeh et al. (36)	2006–10	Persian	Ahvaz	AJUMS	13	Number of physicians, nurses, and beds	Occupied bed day, number of surgeries, outpatients, inpatients, mean patient stay	VRS, input-oriented
18	Akbari et al. (37)	2005–08	Persian	Tabriz	TBZMED	20	Number of physicians and others, beds, and hospital costs	Number of patient admissions and surgeries, bed occupancy rate	VRS, input-oriented
19	Azar et al. (38)	2009–11	Persian	Tehran	TUMS	22	Number of beds, physicians, paramedics, and others	Number of outpatients, emergencies, inpatients, and surgeries, bed occupancy rate	VRS, input-oriented
20	Safi Aryan et al. (39)	2009	Persian	Hamadan	UMSHA	16	Number of beds, physicians, nurses, and others	Number of surgeries and outpatients, bed occupancy rate, mean patient stay, inpatient bed stay	VRS, input-oriented
21	Kazemi et al. (40)	2006–08	Persian	East of Iran	Medical Universities, SSO	11	Number of beds and all employees	Occupied bed day, outpatient admission	VRS, input-oriented
22	Raeisian et al. (41)	2007–11	Persian	Ahvaz	AJUMS & SSO, Private & Charity	8	Number of beds, physicians, nurses, and others	Number of patients and surgeries, bed occupancy rate	VRS, input-oriented
23	Raeisian et al. (41)	2007–11	Persian	Ahvaz	AJUMS & SSO, Private & Charity	8	Number of beds, physicians, nurses, and others	Number of patients and surgeries, bed occupancy rate	VRS, input-oriented
24	Mohebifar et al. (42)	2006–10	Persian	Guilan	GUMS	19	Number of beds, physicians, nurses, and others	Number of outpatients, inpatients, surgeries, and inpatient days	VRS, input-oriented
25	Fazeli et al. (43)	2009–11	Persian	Ilam	MEDILAM	9	Number of beds, physicians, and others	Number of clinical, paraclinical, and outpatient services	Input-oriented
26	Fazeli et al. (43)	2009–13	Persian	Ilam	MEDILAM	9	Number of beds, physicians, and others	Number of clinical, paraclinical, and outpatient services	Input-oriented
27	Mahfoozpor et al. (44)	2013–14	Persian	Tehran	SBMU	10	Number of physicians and nurses	Number of discharged patients	VRS, input-oriented
28	Mahfoozpor et al. (44)	2013–14	Persian	Tehran	SBMU	10	Number of physicians and nurses	Surgery room function	VRS, input-oriented
29	Mahfoozpor et al. (44)	2013–14	Persian	Tehran	SBMU	10	Number of physicians and nurses	Number of discharged patients	VRS, input-oriented
30	Mahfoozpor et al. (44)	2013–14	Persian	Tehran	SBMU	10	Number of physicians and nurses	Surgery room function	VRS, input-oriented
31	Ghasemi et al. (45)	2005–11	Persian	Kermanshah	KUMS	7	Number of beds, physicians, nurses, and others	Number of outpatients, inpatients, occupied bed days, and surgeries	VRS, input-oriented
32	Firouzi et al. (46)	NA	Persian	Tehran	TUMS	40	Number of beds, physicians, paramedics, some costs	Number of contracted insurances, access to emergency, confront with hospital infections, anesthesia problems, employee consent, bed occupancy rate, employee to bed ratio	VRS, input-oriented
33	Amozadeh et al. (47)	2012, 13, 15	Persian	Mazandaran and Babul	Mazandaran & Babol UMS	21	Number of beds, physicians, nurses, and others	Number of emergencies, outpatients, and surgeries	VRS, input-oriented
34	Youzi et al. (48)	2016	Persian	Tehran	TUMS	21	Number of beds, physicians, and nurses	Percentage of active beds, bed occupancy rate, mean stay, and bed turnover	VRS, input-oriented
35	Lotfi et al. (49)	2007–2011	English	Ahvaz	Affiliated and non-affiliated with AJUMS	16	Number of beds, physicians, nurses, and others	Bed occupancy rate, number of patients and operations	Input-oriented
36	Nabilou et al. (9)	2009–2014	English	Tehran	TUMS	17	Number of beds, nurses, physicians, and others	Number of outpatient admission, occupied bed days, surgical operations	Input-oriented, variable return to scale
37	Rezapour et al. (50)	2009–2012	English	Tehran	IUMS & TUMS	19	Human resources, capital resources	Number of inpatients and admissions and inpatient bed occupancy rate	VRS, input-oriented
38	Torabipour et al. (51)	2007–2010	English	Ahvaz	University, Charity, Private	12	Number of nurses, beds, and physicians	Number of outpatients and inpatients, mean hospital stay, number of major operations	Input-oriented
39	Kiadaliri et al. (19)	2006	English	Ahvaz	AJUMS	19	Human resources, number of beds	Number of outpatient and inpatient visits, number of surgeries and percentage of occupied beds	VRS, input-oriented
40	Nabilou et al. (52)	2013–2014	Persian	Urmia	UMSU	23	Number of nurses, physicians, beds, and others	Number of discharges, surgeries, and bed occupancy percentage	VRS, input-oriented
41	Rezaei et al. (53)	2007–2011	English	Kurdistan	MUK	12	Number of beds, nurses, physicians, and others	Number of inpatient admissions and occupied bed days	VRS, input-oriented
42	Goudarzi et al. (54)	2001–07	Persian	Lorestan	LUMS	13	Number of beds, nurses, physicians, and others	Number of outpatients, inpatients, surgeries, bed days, and bed occupancy rate	VRS, input-oriented
43	Askari et al. (55)	2001–11	English	Yazd	SSU	13	Number of beds, nurses, physicians, and non-clinical staff	Number of admissions and surgeries, bed occupancy percentage	VRS, input-oriented
44	Sabermahani et al. (56)	2011	English	Kerman	KMU	13	Full-time physicians and nurses, administrative personnel	Number of outpatient clients, surgeries, and beds per day	VRS, input-oriented
45	Jahangiri et al. (11)	2011–13	Persian	Arak	IAU-ARAK	31	Number of day-beds, working days, physicians, and other staff	Number of admissions, surgeries, child birth, and inpatient days	CRS, input-oriented
46	Najafi et al. (57)	2001–06	Persian	Ardabil	TUMS	10	Number of beds and physicians	Number of admissions and inpatient beds	VRS, input-oriented
47	Hatam et al. (58)	2006–2008	Persian	Iran	SUMS	18	Number of beds and all full-time staff, hospital budget	Bed-day, active to fixed bed, patient mean stay, bed turnover, death, and costs	CRS, input-oriented
48	Rezapour et al. (50)	1998–07	Persian	Qazvin	QUMS	4	Number of beds, physicians, nurses, and others	Number of discharges, surgeries, admissions, emergencies, bed turnover, patient days	VRS, input-oriented
49	Hadian et al. (59)	2006–11	Persian	Tehran	IUMS & TUMS	19	Number of beds, nurses, physicians, and others	Number of outpatient admissions, inpatient days, occupied bed days, surgeries	VRS, input-oriented
50	Mehrtak et al. (60)	NA	English	E. Azarbaijan	IUMS	18	Number of beds, physicians, and nurses	Number of discharges, surgeries, bed occupancy rate	VRS, input-oriented

*TUMS, Tehran University of Medical Sciences; IUMS, Iran University of Medical Sciences; SBMU, Shaheed Beheshti University of Medical Sciences; SSO, Social Security Organization; MUI, Isfahan University of Medical Sciences; SSU, Yazd University of Medical Sciences; TUMS, Tehran University of Medical Sciences; MHH, Ministry of Health' hospitals; MUQ, Qom university of Medical Sciences; MUMS, Mashhad University of Medical Sciences; UMSU, Urmia University of Medical Sciences; AJUMS, Ahvaz Jundishapour University of Medical Sciences; TBZMED, Tabriz University of Medical Sciences; UMSHA, Hamedan University of Medical Sciences; GUMS, Guilan University of Medial Sciences; MEDILAM, Ilam University of Medical Sciences; KUMS, Kermanshah University of Medical Sciences; MUK, Kurdistan University of Medical Sciences; LUMS, Lorestan University of Medical Sciences; KMU, Kerman University of Medical Sciences; IAU-ARAK, Islamic Azad University Branch of Arak; SUMS, Shiraz University of medical sciences; QUMS, Qazvin University of Medical Sciences*.

The results indicated that there was heterogeneity in studies related to technical efficiency (heterogeneity chi^2^ = 156, p < 0.001), managerial efficiency (heterogeneity chi^2^ = 79.58, p < 0.001), and scale efficiency (heterogeneity chi^2^ = 67.22, p < 0.001). I^2^ index in technical and managerial efficiency was higher than 50%, which indicates high heterogeneity between the studies. This index was lower than 50% for scale efficiency.

Study publication error using Egger's test indicated that there was publication bias in technical and managerial efficiencies (P < 0.001), but there was no publication bias in scale efficiency (*p* = 0.19). Table 2 displays the results of Egger's testing for the three types of efficiencies. Begg's test indicated that there was no publication bias in the three types of efficiencies (*P* < 0.001).

**Table 2:** Egger's test for small-study effects to examine the publication bias.

		**Coefficient**	**S.E**.	***P*-value**	**95% confidence interval**	**Test of H0: no small-study effects**
Technical efficiency	Slope	0.56	0.00	0.000	(0.40, 0.73)	*P* = 0.005
	Bias	−1.01	0.34	0.005	(0.32, 1.70)	
Managerial efficiency	Slope	1.00	0.00008	0.000	(0.99, 1.00)	*p* < 0.001
	Bias	−1.22	0.26	0.000	(−1.76, −0.68)	
Economics of scale efficiency	Slope	1.00	0.00009	<0.001	(0.99, 1.00)	*p* < 0.001
	Bias	−1.28	0.19	<0.001	(−1.67, −0.0.90)	

**Figure 2:**
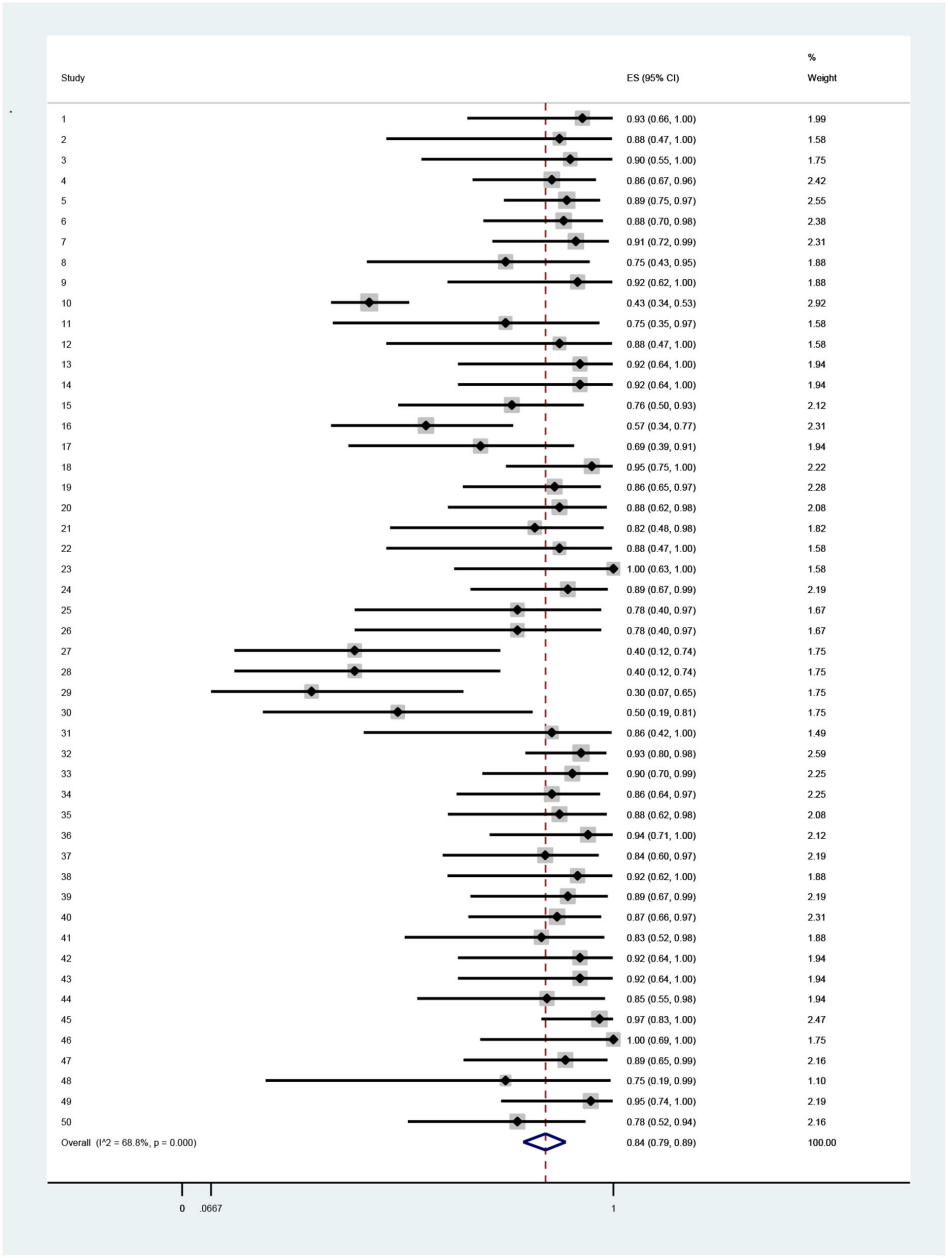
Forest plot of estimates and 95% confidence intervals of the technical efficiency among Iranian hospitals.

**Figure 3:**
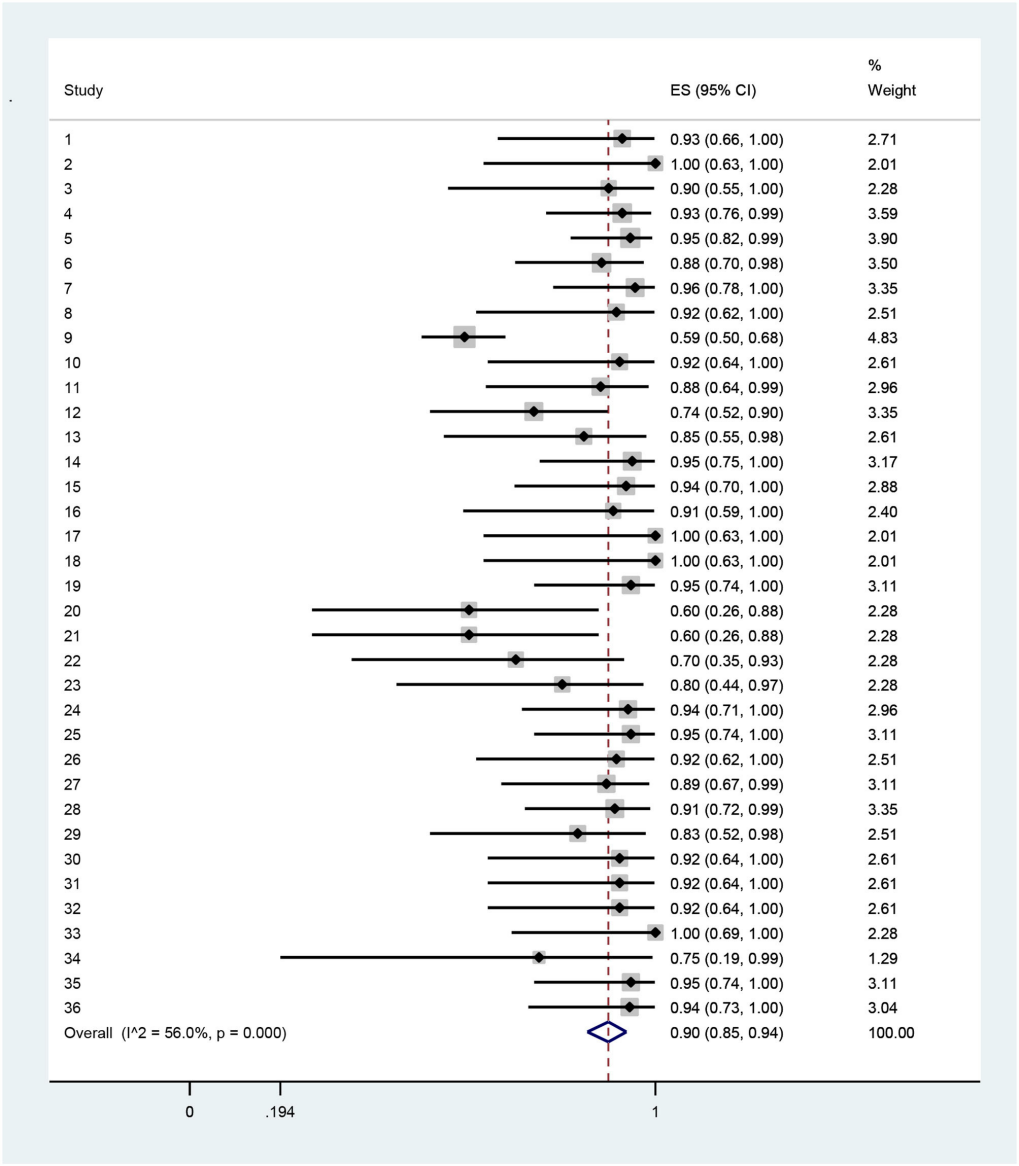
Forest plot of estimates and 95% confidence intervals of the managerial efficiency among Iranian hospitals.

**Figure 4:**
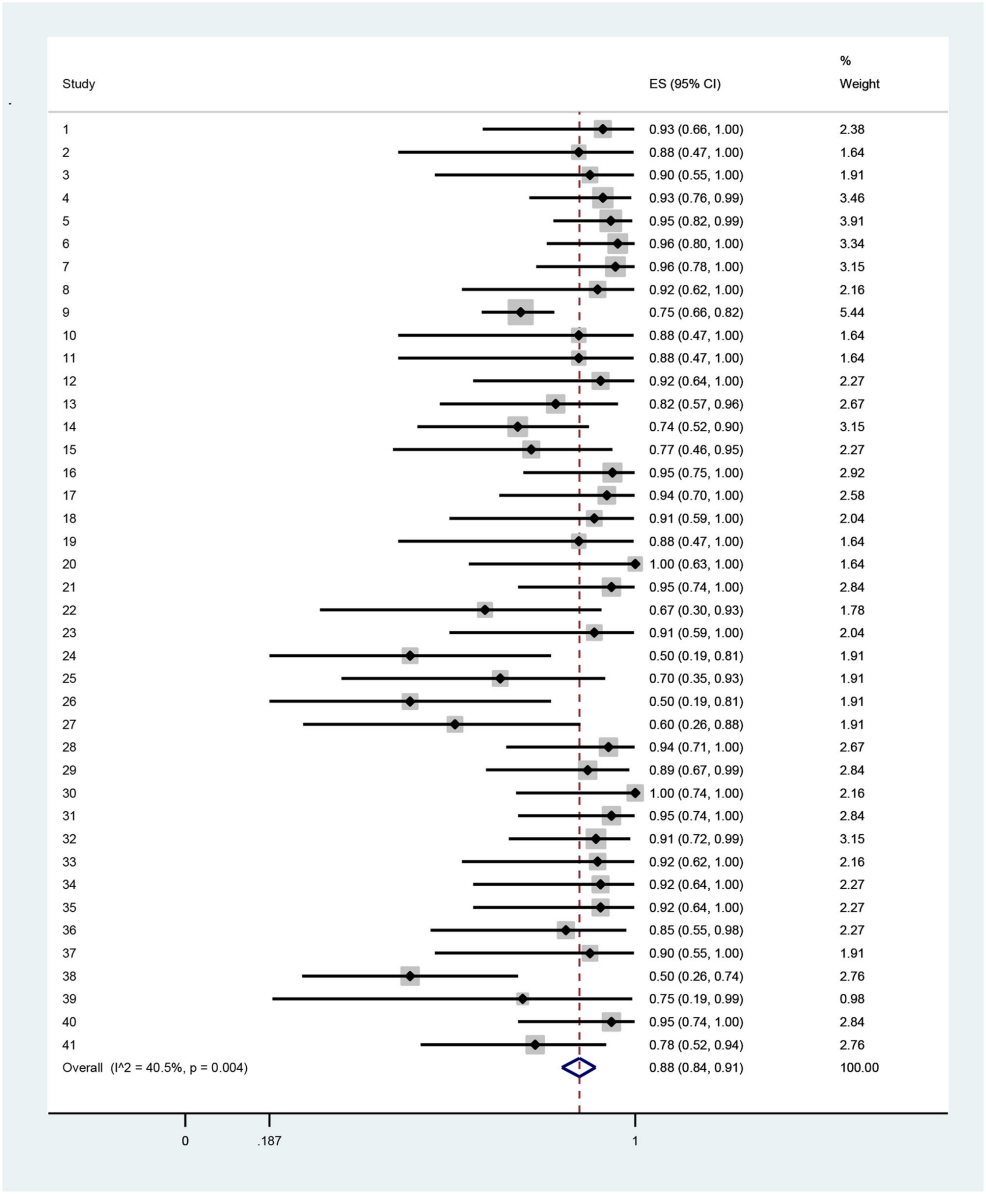
Forest plot of estimates and 95% confidence intervals of the economics of scale efficiency among Iranian hospitals.

The results indicated that technical efficiency of Iranian hospitals had high variation, so that it ranged from 0.34 in the Mahfoozpor et al. study to 1 in Raeisian et al. and Najafi et al. On the basis of random effects modeling, random pooled estimation of hospital technical efficiency was 0.84 (95% CI = 0.52, 0.78) (Table 3, Figure 2). The managerial efficiency of Iranian hospitals was between 0.59 in the Aboulhalaj et al. study and 1 in studies of Joshan et al., Raeisian et al., and Najafi et al. Random pooled estimation of managerial efficiency of Iranian hospitals was 0.90 (95% CI = 0.85,0.94) (Table 4, Figure 3). The lowest amount of scale efficiency (0.52) was in the Mahfoozpor et al. study and the highest (1) was in the Raeisian et al. and Torabipour et al. studies. Random pool estimation of scale efficiency for Iranian hospitals was 0.88 (95%CI = 0.84, 0.91) (Table 5, Figure 4). The results of technical, managerial, and scale efficiencies are presented in Tables 2, 4, 5, respectively. In addition, the forest plots for technical, managerial, and scale efficiencies are presented in Figures 1–3, respectively.

**Table 3:** The results of pool estimation for technical efficiency among Iranian hospitals.

**Study**	**Authors**	**Estimation**	**95% confidence intervals**	**Weight**
1	Joshan et al. (24)	0.93	(0.66, 1)	1.99
2	Joshan et al. (24)	0.88	(0.47, 1)	1.58
3	Joshan et al. (24)	0.9	(0.55, 1)	1.75
4	Sepehrdost et al. (25)	0.86	(0.67, 0.96)	2.42
5	Sepehrdost et al. (25)	0.89	(0.75, 0.97)	2.55
6	Ghaderi et al. (26)	0.88	(0.7, 0.98)	2.38
7	Karimi et al. (27)	0.91	(0.72, 0.99)	2.31
8	Alimohammadi Ardakani et al. (28)	0.75	(0.43, 0.95)	1.88
9	Pourreza et al. (29)	0.92	(0.62, 1)	1.88
10	Aboulhalaj et al. (30)	0.43	(0.34, 0.53)	2.92
11	Salehzade et al. (31)	0.75	(0.35, 0.97)	1.58
12	Salehzade et al. (31)	0.88	(0.47, 1)	1.58
13	Asadi et al. (32)	0.92	(0.64, 1)	1.94
14	Askari et al. (33)	0.92	(0.64, 1)	1.94
15	Ilbeigi et al. (34)	0.76	(0.5, 0.93)	2.12
16	Rahimi et al. (35)	0.57	(0.34, 0.77)	2.31
17	Najarzadeh et al. (36)	0.69	(0.39, 0.91)	1.94
18	Akbari et al. (37)	0.95	(0.75, 1)	2.22
19	Azar et al. (38)	0.86	(0.65, 0.97)	2.28
20	Safi Aryan et al. (39)	0.88	(0.62, 0.98)	2.08
21	Kazemi et al. (40)	0.82	(0.48, 0.98)	1.82
22	Raeisian et al. (41)	0.88	(0.47, 1)	1.58
23	Raeisian et al. (41)	1	(0.63, 1)	1.58
24	Mohebifar et al. (42)	0.89	(0.67, 0.99)	2.19
25	Fazeli et al. (43)	0.78	(0.4, 0.97)	1.67
26	Fazeli et al. (43)	0.78	(0.4, 0.97)	1.67
27	Mahfoozpor et al. (44)	0.4	(0.12, 0.74)	1.75
28	Mahfoozpor et al. (44)	0.4	(0.12, 0.74)	1.75
29	Mahfoozpor et al. (44)	0.3	(0.07, 0.65)	1.75
30	Mahfoozpor et al. (44)	0.5	(0.19, 0.81)	1.75
31	Ghasemi et al. (45)	0.86	(0.42, 1)	1.49
32	Firouzi Jahantigh et al. (46)	0.93	(0.8, 0.98)	2.59
33	Amozadeh et al. (47)	0.9	(0.7, 0.99)	2.25
34	Youzi et al. (48)	0.86	(0.64, 0.97)	2.25
35	Lotfi et al. (49)	0.88	(0.62, 0.98)	2.08
36	Nabilou et al. (9)	0.94	(0.71, 1)	2.12
37	Rezapour et al. (50)	0.84	(0.6, 0.97)	2.19
38	Torabipour et al. (51)	0.92	(0.62, 1)	1.88
39	Ahmad Kiadaliri et al. (19)	0.89	(0.67, 0.99)	2.19
40	Nabilou et al. (52)	0.87	(0.66, 0.97)	2.31
41	Rezaei et al. (53)	0.83	(0.52, 0.98)	1.88
42	Goudarzi et al. (54)	0.92	(0.64, 1)	1.94
43	Askari et al. (55)	0.92	(0.64, 1)	1.94
44	Sabermahani et al. (56)	0.85	(0.55, 0.98)	1.94
45	Jahangiri et al. (11)	0.97	(0.83, 1)	2.47
46	Najafi et al. (57)	1	(0.69, 1)	1.75
47	Hatam et al. (58)	0.89	(0.65, 0.99)	2.16
48	Rezapour et al. (50)	0.75	(0.19, 0.99)	1.1
49	Hadian et al. (59)	0.95	(0.74, 1)	2.19
50	Mehrtak et al. (60)	0.78	(0.52, 0.94)	2.16
Random pooled estimation		0.84	(0.78, 0.52)	100

*Heterogeneity chi^2^ = 156.97 (d.f. = 49) p = 0.000*.

*I^2^ (variation in ES attributable to heterogeneity) = 68.78%*.

*Estimate of between-study variance Tau^2^ = 0.12*.

**Table 4:** The results of pool estimation for managerial efficiency among Iranian hospitals.

**Study**	**Authors**	**Estimation**	**95% confidence intervals**	**Weight**
1	Joshan et al. (24)	0.93	(0.66, 1)	2.71
2	Joshan et al. (24)	1	(0.63, 1)	2.01
3	Joshan et al. (24)	0.9	(0.55, 1)	2.28
4	Sepehrdost et al. (25)	0.93	(0.76, 0.99)	3.59
5	Sepehrdost et al. (25)	0.95	(0.82, 0.99)	3.9
6	Ghaderi et al. (26)	0.88	(0.7, 0.98)	3.5
7	Karimi et al. (28)	0.96	(0.78, 1)	3.35
8	Pourreza et al. (29)	0.92	(0.62, 1)	2.51
9	Aboulhalaj et al. (30)	0.59	(0.5, 0.68)	4.83
10	Askari et al. (33)	0.92	(0.64, 1)	2.61
11	Ilbeigi et al. (34)	0.88	(0.64, 0.99)	2.96
12	Rahimi et al. (35)	0.74	(0.52, 0.9)	3.35
13	Najarzadeh et al. (36)	0.85	(0.55, 0.98)	2.61
14	Akbari et al. (37)	0.95	(0.75, 1)	3.17
15	Safi Aryan et al. (39)	0.94	(0.7, 1)	2.88
16	Kazemi et al. (40)	0.91	(0.59, 1)	2.4
17	Raeisian et al. (41)	1	(0.63, 1)	2.01
18	Raeisian et al. (41)	1	(0.63, 1)	2.01
19	Mohebifar et al. (42)	0.95	(0.74, 1)	3.11
20	Mahfoozpor et al. (44)	0.6	(0.26, 0.88)	2.28
21	Mahfoozpor et al. (44)	0.6	(0.26, 0.88)	2.28
22	Mahfoozpor et al. (44)	0.7	(0.35, 0.93)	2.28
23	Mahfoozpor et al. (44)	0.8	(0.44, 0.97)	2.28
24	Nabilou et al. (9)	0.94	(0.71, 1)	2.96
25	Rezapour et al. (50)	0.95	(0.74, 1)	3.11
26	Torabipour et al. (51)	0.92	(0.62, 1)	2.51
27	Ahmad Kiadaliri et al. (19)	0.89	(0.67, 0.99)	3.11
28	Nabilou et al. (52)	0.91	(0.72, 0.99)	3.35
29	Rezaei et al. (53)	0.83	(0.52, 0.98)	2.51
30	Goudarzi et al. (54)	0.92	(0.64, 1)	2.61
31	Askari et al. (55)	0.92	(0.64, 1)	2.61
32	Sabermahani et al. (56)	0.92	(0.64, 1)	2.61
33	Najafi et al. (57)	1	(0.69, 1)	2.28
34	Rezapour et al. (50)	0.75	(0.19, 0.99)	1.29
35	Hadian et al. (59)	0.95	(0.74, 1)	3.11
36	Mehrtak et al. (60)	0.94	(0.73, 1)	3.04
Random pooled estimation		0.9	(0.85, 0.94)	100

*Heterogeneity chi^2^ = 79.58 (d.f. = 35), p = 0.000*.

*I^2^ (variation in ES attributable to heterogeneity) = 56.02%*.

*Estimate of between-study variance Tau^2^ = 0.07*.

*Test of ES = 0: z = 34.96, p = 0.00*.

**Table 5:** The results of pool estimation for economies of scale efficiency among Iranian hospitals.

**Study**	**Authors**	**Estimation**	**95%confidence Intervals**	**Weight**
1	Joshan et al. (24)	0.93	(0.66, 1)	2.38
2	Joshan et al. (24)	0.88	(0.47, 1)	1.64
3	Joshan et al. (24)	0.9	(0.55, 1)	1.91
4	Sepehrdost et al. (25)	0.93	(0.76, 0.99)	3.46
5	Sepehrdost et al. (25)	0.95	(0.82, 0.99)	3.91
6	Ghaderi et al. (26)	0.96	(0.8, 1)	3.34
7	Karimi et al. (27)	0.96	(0.78, 1)	3.15
8	Poureza et al. (29)	0.92	(0.62, 1)	2.16
9	Aboulhalaj et al. (30)	0.75	(0.66, 0.82)	5.44
10	Salehzade et al. (31)	0.88	(0.47, 1)	1.64
11	Salehzade et al. (31)	0.88	(0.47, 1)	1.64
12	Askari et al. (33)	0.92	(0.64, 1)	2.27
13	Ilbeigi et al. (34)	0.82	(0.57, 0.96)	2.67
14	Rahimi et al. (35)	0.74	(0.52, 0.9)	3.15
15	Najarzadeh et al. (36)	0.77	(0.46, 0.95)	2.27
16	Akbari et al. (37)	0.95	(0.75, 1)	2.92
17	Safi Aryan et al. (39)	0.94	(0.7, 1)	2.58
18	Kazemi et al. (40)	0.91	(0.59, 1)	2.04
19	Raeisian et al. (41)	0.88	(0.47, 1)	1.64
20	Raeisian et al. (41)	1	(0.63, 1)	1.64
21	Mohebifar et al. (42)	0.95	(0.74, 1)	2.84
22	Fazeli et al. (43)	0.67	(0.3, 0.93)	1.78
23	Fazeli et al. (43)	0.91	(0.59, 1)	2.04
24	Mahfoozpor et al. (44)	0.5	(0.19, 0.81)	1.91
25	Mahfoozpor et al. (44)	0.7	(0.35, 0.93)	1.91
26	Mahfoozpor et al. (44)	0.5	(0.19, 0.81)	1.91
27	Mahfoozpor et al. (44)	0.6	(0.26, 0.88)	1.91
28	Nabilou et al. (9)	0.94	(0.71, 1)	2.67
29	Rezapour et al. (50)	0.89	(0.67, 0.99)	2.84
30	Torabipour et al. (51)	1	(0.74, 1)	2.16
31	Ahmad Kiadaliri et al. (19)	0.95	(0.74, 1)	2.84
32	Nabilou et al. (9)	0.91	(0.72, 0.99)	3.15
33	Rezaei et al. (53)	0.92	(0.62, 1)	2.16
34	Goudarzi et al. (54)	0.92	(0.64, 1)	2.27
35	Askari et al. (55)	0.92	(0.64, 1)	2.27
36	Sabermahani et al. (56)	0.85	(0.55, 0.98)	2.27
37	Najafi et al. (57)	0.9	(0.55, 1)	1.91
38	Hatam et al. (58)	0.5	(0.26, 0.74)	2.76
39	Rezapour et al. (50)	0.75	(0.19, 0.99)	0.98
40	Hadian et al. (59)	0.95	(0.74, 1)	2.84
41	Mehrtak et al. (60)	0.78	(0.52, 0.94)	2.76
Random pool estimation		0.88	(0.84, 0.91)	100

*Heterogeneity chi^2^ = 67.22 (d.f. = 40) p = 0.00*.

*I^2^ (variation in ES attributable to heterogeneity) = 40.5%*.

*Test of ES = 0: z = 40.93 p = 0.00*.

Sub-group analysis based on study year indicated that random pool estimation of technical efficiency of Iranian hospitals for 2011 and before and after 2011 was 0.86 (95% CI = 0.80, 0.91) and 0.78 (95%CI = 0.64, 0.89), respectively. The status of managerial efficiency for 2011 and before was better than after 2011 (random pool estimation equal to 0.91, compared to 0.86). Random pool estimation of scale efficiency for 2011 and before was 0.90 (95%CI = 0.86, 0.93), while random pool estimation of scale efficiency for after 2011 was 0.74 which is lower (95%CI = 0.61, 0.86) (Table 6).

**Table 6:** The random pool estimation of technical, managerial, and economics of scale efficiencies among Iranian hospitals by time of studies.

	**Subgroup**	**Estimation**	**95% confidence intervals**	**Weight**	**Test(s) of heterogeneity**		**Random Test for heterogeneity between sub-groups:**
					I^2^^**^	***P*-value**	***P*-value**
Technical efficiency	2011 and before	0.86	(0.80, 0.91)	75.97	66.80%	0.000	0.23
	After 2011	0.78	(0.64, 0.89)	24.03	75.55%	0.000	
Managerial efficiency	2011 and before	0.91	(0.86, 0.95)	77.89	60.74%	0.000	0.27
	After 2011	0.86	(0.75, 0.94)	22.11	39.89%	0.100	
Economics of scale efficiency	2011 and before	0.90	(0.86, 0.93)	83.67	32.79%	0.040	0.01
	After 2011	0.74	(0.61, 0.86)	16.33	35.26%	0.150	

** *I^2^, the variation in ES attributable to heterogeneity*.

## Discussion

The assessment of hospital efficiency provides the groundwork to assess their performance and increase productivity when using limited resources. One of the ways of assessing allocated resources to obtain specified goals is efficiency studies. In summary, efficiency means using resources to their maximum to produce goods and services (61).

This is the first systematic review and meta-analysis study regarding assessment of the efficiency of Iranian hospitals in terms of its subcategories namely technical, managerial, and scale efficiencies. Different methods have been used to assess Iranian hospital efficiency such as DEA, Pabon-Lasso, and Stochastic Frontier Analysis (SFA) in past decades (21). In this regard, as this study indicates, the DEA method is the most widely applied method to assess hospital efficiency (19).

Our findings showed that the random pool estimations of technical, managerial, and economics of scale efficiency were 0.87, 0.9, and 0.88, respectively. This finding indicates that the resources of the studied hospitals in Iran have been used in an inefficient way. One idea about hospital efficiency is that the expectation from hospitals to work efficiently is far from reality. The reasoning for this claim is the economic theory of firms that declare the hospitals cannot work at full efficiency because of uncertainty in costs and prices of services that they provide. In summary, lack of information on costs and prices is one of the main factors that has a negative effect on hospital efficiency (6, 62).

Most of the studies were implemented in Tehran province (13 studies). Four studies investigated the efficiency of hospitals across all provinces of Iran. However, some provinces such as Sistan and Baluchistan had no individual reports about the efficiency of their hospitals. Therefore, there is an information gap for health policymakers and hospital managers in this field.

As the results indicated, most of the researchers tended to perform analyzes through the input-oriented method, because inputs are in the control of hospital managers, so that by creating changes in the inputs can change the rate of outputs to the desired extent. However, it is suggested that private and for-profit hospitals are excluded from this rule, because the managers of these type of hospitals want to maximize outputs and, as a result, hospital profits (63).

Human and capital resources such as the number of nurses and physicians and the number of beds were the main inputs in all included studies. Number of surgeries, outpatient admissions, inpatient admissions, bed days, and bed occupancy rate were the most frequent outputs considered in the studies to estimate the efficiency of hospitals. Today, the management of all resources, especially human resources in the health care industry is recognized as a vital issue for all healthcare organizations (64). Furthermore, better management of human resources is associated with higher patient outcomes without significant increases in the cost of hospitals (65).

The results indicated that most hospital efficiency studies suffer from some weak points. Therefore, the selection of inputs has been performed on the basis of resource review (e.g., previous published articles) not consideration of each hospital situation. Also, the inputs were not weighted, so that the resources with high specialty and expenditure receive the same weight as others. Hospital case mix has not been considered in this hospital efficiency assessment. This leads to low efficiency assessment in hospitals which have the most complicated cases. Lastly, some studies have not considered the data validity and the appropriate ratio of inputs and outputs with the number of hospitals precisely.

The study of Contor VJM and Poh Kl also provides some theoretical and methodological limitations of the DEA method in capturing the full view of efficiency of healthcare centers (66). However, with a suitable study design, the DEA method is among the most important and most applicable methods in the assessment of health system efficiency, especially hospitals (67).

The results indicated that technical, managerial, and scale efficiency of Iranian hospitals after performing HSEP decreased in comparison with before it. A study on Turkey hospitals from 2001 to 2006, which measured the effect of Turkey health sector reform on hospital efficiency to provide policy implications for policymakers, indicated that this reform had increased the efficiency of public hospitals but the efficiency of private hospitals had decreased (68).

As there was no hospital with full efficiency in the study and the increasing trend of health system costs and scarce resources, it is proposed to design and implement a system to monitor efficiency and consumption of resources especially in hospitals. This can help to identify inefficient hospitals and the causes of it. Health policymakers through cost management planning and increasing outputs can pave the way in this regard.

### Strengths and Limitations

This is the first comprehensive systematic review and meta-analysis evaluating efficiency of Iranian hospitals which is applicable for comparison of the efficiency of hospitals before and after HSEP. The methodology adhered to the PRISMA statement (22).

The strength of the study is in performing meta-analysis after the systematic review which has specified the exact amount of technical, managerial, and scale efficiencies of Iranian hospitals. The Cochrane Consumers and Communication Review Group's data extraction template (69) was used to obtain the needed information about the studies included. Nevertheless, the retrieved studies were mainly administered in some easily accessible areas rather than in a balanced distribution all over the country. This limits the generalizability of the results.

## Conclusion

This study indicated that many hospitals are inefficient. This implies that there is considerable room for efficiency improvement in hospitals. Hospital management has a unique role in this regard. Health systems have reformed in spite of increasing access and utilization of patients to the services, but have not considered efficiency improvement in hospitals. So, health policymakers and hospital managers should design and implement some related programs in order to monitor and improve the efficiency of hospitals.

## Data Availability Statement

The original contributions presented in the study are included in the article/supplementary material, further inquiries can be directed to the corresponding author/s.

## Ethics Statement

This systematic review and meta-analysis study was approved by the Ethical Committee of Arak University of Medical Sciences (Ethical Code Number: IR.ARAKMU.REC.1398.044).

## Author Contributions

SA, BK, AK-K, and MD: conception and design of study/review/case series. SA, MA, YS, and AA: acquisition of data. YS, AA, MD, BK, and MA: analysis of collected data. SA, AK-K, YS, MD, AA, and YS: interpretation of data and drafting of paper and/or critical revision. All the authors have read and approved the manuscript to be submitted to BMC Health Services Research.

## Funding

This paper is retrieved from an approved research project. The Deputy of Research of Arak University of Medical Sciences has financially supported this study in different parts of the study including design, data collection, analysis, interpretation, and writing the manuscript (Grant Number: 3382).

## Conflict of Interest

The authors declare that the research was conducted in the absence of any commercial or financial relationships that could be construed as a potential conflict of interest.

## Publisher's Note

All claims expressed in this article are solely those of the authors and do not necessarily represent those of their affiliated organizations, or those of the publisher, the editors and the reviewers. Any product that may be evaluated in this article, or claim that may be made by its manufacturer, is not guaranteed or endorsed by the publisher.

## Abbreviations

HSEP: Health Sector Evolution Plan
CI: confidence interval
DEA: data envelopment analysis
DMUs: decision making units
SSO: Social Security Organization
ISI: Institute for Scientific Information
SID: Scientific Information Database
PRISMA: Preferred Reporting Items for Systematic Reviews and Meta-Analyses.
